# Cysteine protease inhibition by nitrile-based inhibitors: a computational study

**DOI:** 10.3389/fchem.2013.00039

**Published:** 2013-12-27

**Authors:** Matthew G. Quesne, Richard A. Ward, Sam P. de Visser

**Affiliations:** ^1^Manchester Institute of Biotechnology and School of Chemical Engineering and Analytical Science, University of ManchesterManchester, UK; ^2^Oncology iMED Chemistry, AstraZenecaMacclesfield, UK

**Keywords:** enzyme mechanism, enzyme inhibition, DFT, QM/MM

## Abstract

Cysteine protease enzymes are important for human physiology and catalyze key protein degradation pathways. These enzymes react via a nucleophilic reaction mechanism that involves a cysteine residue and the proton of a proximal histidine. Particularly efficient inhibitors of these enzymes are nitrile-based, however, the details of the catalytic reaction mechanism currently are poorly understood. To gain further insight into the inhibition of these molecules, we have performed a combined density functional theory and quantum mechanics/molecular mechanics study on the reaction of a nitrile-based inhibitor with the enzyme active site amino acids. We show here that small perturbations to the inhibitor structure can have dramatic effects on the catalysis and inhibition processes. Thus, we investigated a range of inhibitor templates and show that specific structural changes reduce the inhibitory efficiency by several orders of magnitude. Moreover, as the reaction takes place on a polar surface, we find strong differences between the DFT and QM/MM calculated energetics. In particular, the DFT model led to dramatic distortions from the starting structure and the convergence to a structure that would not fit the enzyme active site. In the subsequent QM/MM study we investigated the use of mechanical vs. electronic embedding on the kinetics, thermodynamics and geometries along the reaction mechanism. We find minor effects on the kinetics of the reaction but large geometric and thermodynamics differences as a result of inclusion of electronic embedding corrections. The work here highlights the importance of model choice in the investigation of this biochemical reaction mechanism.

## Introduction

Proteases are important enzymes in human physiology and involved in terminal protein degradation via a nucleophilic acid-base reaction (Chapman et al., 1997; Otto and Schirmeister, 1997; Madala et al., 2010). They are classified within four different classes that are based on the Lewis base in the catalytic reaction mechanism, namely serine-, cysteine-, aspartate-, and metalloproteases. The former two uses the terminal side chain of a serine or cysteine amino acid as nucleophilic center in the reaction mechanism and are the most abundant proteases in the body. Members of the cysteine protease family include intracellular proteases called cathepsins that take part in hydrolytic cleavage of peptide bonds, apoptosis as well as immune responses. As such, the pathogenesis of microbial infections, arthritis, osteoporosis and cancer require the involvement of cysteine proteases, which makes them an important target of drug development and chemotherapy (Selzer et al., 1999; Palermo and Joyce, 2007; Robinson et al., 2008). In addition, they have been implicated in atherosclerosis-based vascular diseases (Lutgens et al., 2007; Cheng et al., 2011; Ratovitski et al., 2011).

Due to their pharmaceutical interest considerable research has been devoted to establish the mechanism and function of cysteine protease enzymes (Kato et al., 2005; Hang et al., 2006; Shokhen et al., 2011). Furthermore, research into finding suitable inhibitors of the cysteine protease family is paramount (Arkin and Wells, 2004; Rzychon et al., 2004; Drahl et al., 2005; Tyndall et al., 2005; Ehmke et al., 2011). Inhibition can take place via several different methods, including covalent inhibition, blockage or distortion of the catalytic active site via competition with non-covalent inhibitors. One particular class of covalent inhibitors that have been particularly useful for cysteine proteases are nitrile-based compounds (Oballa et al., 2007; MacFaul et al., 2009; Morley et al., 2009; Ehmke et al., 2012). Currently, however, the precise mechanism of the interaction between these inhibitors and active site cysteine is not clear. In order to understand the inhibition process by cysteine proteases, and, especially, its mechanism with inhibitors we decided to do a computational study. The calculations use density functional theory (DFT) on model complexes and quantum mechanics/molecular mechanics (QM/MM) studies on a complete cysteine protease enzyme.

**Figure 1:**
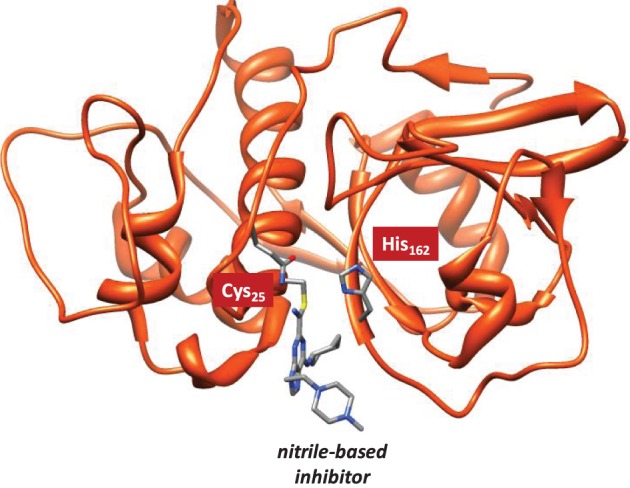
**Extract of the 1U9V pdb structure of cathepsin K with key amino acids and inhibitor highlighted**.

Cysteine proteases bind their substrates in a groove located on the surface of the enzyme, therefore the reaction takes place on a relatively polar region with many interacting hydrogen bonding donor and acceptor groups (Sajid and McKerrow, 2002). A protein-ligand structure of cathepsin K (Altmann et al., 2004) is shown in Figure 1, whereby the nitrile-based inhibitor is covalently linked to a cysteine residue (Cys_25_) in the active site of the protease and within hydrogen bonding distance to a histidine residue (His_162_). It has been hypothesized that this histidine residue donates a proton during the covalent linkage formation between inhibitor and the cysteine residue, but the intricate details of this mechanism remain elusive. The computational study described in this work will address this mechanism and establish the key factors that drive the reaction mechanism as well as the influence of the surrounding protein.

Several groups have studied the catalytic mechanism of cysteine protease with computational techniques, including quantum mechanics/molecular mechanics (QM/MM) (Helten et al., 2004; Ma et al., 2007; Mladenovic et al., 2008a,b,c) and density functional theory (DFT) model complexes and focused on cathepsin B proteases (Shokhen et al., 2009; Shankar et al., 2010). These studies implicated strong influences of the hydrogen bonding network in the substrate/inhibitor binding pocket and led to increased stability of the HisH^+^—Cys^−^ ion pair as compared to its neutral form, although this may change after substrate binding. QM/MM studies on the catalytic mechanism of these cysteine proteases revealed a rate determining acylation step with a barrier of 19.8 kcal mol^−1^ (Ma et al., 2007). These values compare well with other nucleophilic addition reactions in related enzymes, such as sortase A that has a similar active site as cysteine protease and reacts with a similar catalytic machinery (Tian and Eriksson, 2011). So far, few studies have addressed inhibitor binding and the effect of compound reactivity and in particular nitrile-based inhibitors have not been thoroughly studied computationally in this way (Mladenovic et al., 2008c). We therefore initiated this study to understand the covalent mechanism of this chemotype through its binding to cathepsin K.

**SCHEME 1:**
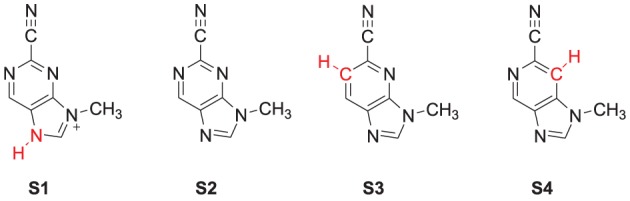
**Nitrile-based inhibitor templates investigated in this study**.

The specific mechanism of how nitrile-based inhibitors interact with cathepsin K is not known, it is however clear that these inhibitors bind tightly within the active site and inhibit via the reversible formation of a covalent bond (Helten et al., 2004). To find out, how structural changes to these inhibitors affect their inhibition, we modeled the reaction of cathepsin K with four inhibitor scaffolds (Scheme 1): S1 and S2 are the protonated and neutral purine-based nitriles, whereas in S3 and S4 the nitrogen atoms of the six-membered ring were replaced by carbon. The purpose of investigating S3 and S4 is because studies have shown such a change results in a significant reduction in the reactivity of the nitrile functionality (Altmann et al., 2004). As the reaction takes place on the surface of the enzyme, and the interactions between the inhibitors and protein affect the reaction we have opted for two sets of calculations. In the first set we take a model of the substrate binding-site and reaction position and calculate the mechanism with density functional theory. In a subsequent set of calculations we use quantum mechanics/molecular mechanics (QM/MM) on the complete enzyme. As we show here, there are dramatic differences in substrate activation between the two models that highlight the importance of the full QM/MM model in the calculations.

## Methods

The studies presented in this work use density functional theory (DFT) as well as quantum mechanics/molecular mechanics (QM/MM) methods as implemented in the *Jaguar* and *Gaussian*-03 program packages (Frisch et al., 2004; Jaguar, 2009). We used two approaches to investigate the mechanism of cysteine protease inhibition: (*i*) DFT calculations on a model complex that represents the active species and key hydrogen bonding interactions, so-called QM-cluster calculations, and (*ii*) Full QM/MM on the complete enzyme. In the past we used the QM-cluster methodology extensively to describe the mechanistic features of important enzymes for human health, for example of the cytochromes P450, (Shaik et al., 2005; de Visser, 2010; Kumar et al., 2010) cysteine dioxygenase (Aluri and de Visser, 2007) and taurine/α-ketoglutarate dependent dioxygenase (de Visser, 2006). Although these QM-cluster calculations in many cases were able to reproduce experimentally determined product distributions, kinetic isotope effects and rate constants as well as spectroscopic features of key stable intermediates (Kumar et al., 2005; Vardhaman et al., 2011, 2013a,b), we cannot be certain that the methodology will also work on an enzyme such as a cysteine protease. In particular, QM/MM studies on these enzymatic systems showed that in many cases the active features were sufficient to describe the enzyme accurately (Godfrey et al., 2008; Porro et al., 2009; Kumar et al., 2011b), but in cases where the active species has close lying electronic states environmental perturbations were shown to change the electronic properties of the reactant and consequently the reactivity patterns (Ogliaro et al., 2000; de Visser et al., 2002; Leeladee et al., 2012). We anticipated similar problems in the current system, where strong polar interactions influenced the reaction kinetics, therefore, a combined DFT and QM/MM approach was applied.

### QM-cluster calculations

**SCHEME 2:**
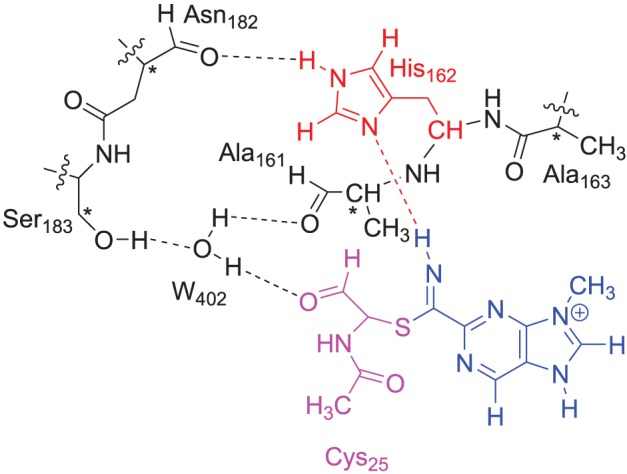
**DFT and QM region of the QM/MM model of cysteine protease used in this study**. Atoms marked with a star were kept fixed in the DFT calculations.

The DFT model complex is based on the key features of the active site of cysteine protease with inhibitor bound as displayed in Scheme 2. Since, the inhibitor is bound on the surface of the enzyme via a range of hydrogen bonding interactions, we included a large proportion of the active site including these key interacting residues. Important hydrogen bonding interactions of the protein with these inhibitor templates, include the interaction with the cysteine residue, the alcohol group of Ser_183_, the peptide chain carbonyl group of Ala_161_, Ala_163_ and Asn_182_, and a bridging water molecule (W_402_). To prevent unnatural changes in the geometry during the optimization procedure, we fixed several carbon atoms of the peptide chains as identified with a star in Scheme 2. However, as geometric constraints often lead to geometry convergence problems and small imaginary frequencies, we kept the number of fixed atoms to a minimum here (Pratter et al., 2013; de Visser et al., 2014). This model has overall charge of +1 and was calculated in the singlet spin state only. Note that the structural constraint had little effect on the geometry optimization and all local minima had real frequencies only. The inhibition of nitriles by cysteine protease was investigated with four different inhibitor molecules as identified with S1, S2, S3 and S4 in Scheme 1 above. S1 and S2 are based on the inhibitor bound in the 1U9V pdb file and differ by the protonation state of the nitrogen atom of the five-membered ring. As a consequence the model with substrate S1 has overall charge +1, whereas the model with substrate S2 is neutral. In addition, we investigated two substrates S3 and S4, where one of the nitrogen atoms of the pyridine ring was replaced by a CH group. These structures are expected to have a lower intrinsic reactivity and differ in their hydrogen bond acceptor patterns. It has been shown (Ehmke et al., 2012) that molecules with high electrophilicity inhibit cysteine protease better than those with lower electrophilicity. In particular, compounds, such as S2, which contain a pyrimidine, were found to inhibit more effectively than compounds based on the S3 and S4 templates that use a pyridine instead.

Following previous experience with DFT on nucleophilic addition reactions (de Visser, 2012), we use the hybrid density functional method B3LYP (Becke, 1993; Lee et al., 1998) in combination with a 6-31G* basis set, BS1, for geometry optimizations and frequency calculations. We performed a full geometry optimization followed by an analytical frequency. To test the effect of the basis set on the energetics we ran single point B3LYP/6-311++G^**^ calculations, basis set BS2. For a selection of reactions we also tested the effect of solvent using the polarized continuum model as implemented in *Jaguar* with a dielectric constant of ϵ = 5.7 and a probe radius of 2.66 Å.

### QM/MM set-up

In a second set of calculations we carried out QM/MM calculations starting from the inhibitor bound structure of cathepsin K: 1U9V pdb structure (Altmann et al., 2004). We used well tested QM/MM procedures, which we applied to the catalytic reaction mechanisms of heme and non-heme iron enzymes previously (Godfrey et al., 2008; Porro et al., 2009; Kumar et al., 2011b; Quesne et al., 2014). The work started out from the inhibitor-bound enzyme monomer as deposited as the 1U9V crystal structure, which we initially updated to include all missing heavy atoms of the amino acids using the MOE program package (MOE, 2008). Subsequently, we used these starting coordinates to add hydrogen atoms to the heavy atoms with the PDB2PQR program package (Dolinsky et al., 2007). All amino acids were then protonated according to the usual p*K*_*a*_ conventions (Dolinsky et al., 2007) at pH = 7 and analyzed with the Propka program package; this resulted in a structure with all aspartate and glutamate amino acids in their deprotonated forms and all arginine and lysine amino acids as protonated. The peptide chain contains two histidine amino acids and upon visual inspection it was decided to take His_162_ as doubly protonated, whereas His_177_ as singly protonated on atom Nϵ only. This gave us a system that is overall charge neutral. Subsequently, our chemical system was solvated in a sphere where the protein was extended with a water layer with a diameter of 10 Å using periodic boundary conditions. We repeated the solvation procedure in several steps where we attempted to add further water molecules to the system after each step and kept repeating this process until few water molecules could be added. This resulted in a chemical system with a total of 9580 atoms. Note, that the structure is energy minimized after each water addition step until a threshold of minimum number of water molecules added was reached. This system was then equilibrated and subjected to a molecular mechanics minimization using the FF94 force field (Wang et al., 2004) and then gradually heated to above room temperature (298 K) conditions using a heating procedure. During the equilibration the water accessible areas of the protein were comprehensibly solvated and the protein unfolded into a more relaxed structure using a dynamical protocol built into the MOE software package (MOE, 2008). The equilibration was performed in a consecutive set of minimizations starting with restrained protein and ligand and followed by an fully unrestrained calculation. Subsequently, the chemical system was gradually heated from 0 to 300 K with restrained ligand and protein. Finally, an NVT molecular dynamics simulation was done for 500 ps in steps of 0.001 ps until a temperature of 300 K was reached. In order to parameterize and obtain a topology for our purine-based nitrile inhibitor the antechamber component of the AMBER 10 package was used (Cornell et al., 1995; Tao and Schlegel, 2010). In this particular case, for each atom and bond type specific topology and parameters were identified based on known residue values.

### QM/MM calculations

We then selected a QM region that contained the same model as displayed in Scheme 2 for the DFT model calculations. The QM region was overall charge neutral and was calculated in the closed-shell singlet spin state only. The ONIOM approach (Vreven et al., 2006). was used for the QM/MM calculations with the B3LYP/6-311G method describing the QM region. We tested several force fields for the description of the MM region including the UFF/ZDO and Amber methods (Rappé et al., 1992; Cornell et al., 1995). The link-atom approach (Singh and Kollman, 1986) describes the division between the QM and MM regions as hydrogen link-atoms. All structures and energies reported here were fully optimized (without constraints) using DFT/AMBER in Gaussian-03, whereby all atoms within a radius of 8Å from the reaction center were free to move during the optimization and the rest was fixed in its position throughout the QM/MM calculation. As frequency calculations were impossible on the full QM/MM structure in Gaussian, we extracted the QM region from the optimized QM/MM geometries, added hydrogen atoms and ran an analytical frequency calculation on the QM region only. To prevent excessive small imaginary frequencies in structures, we reoptimized the hydrogen atoms before running the frequency calculation, however, none of the local minima discussed here had any imaginary frequencies. As will be shown in the results section the differences in ΔE and ΔE+ZPE energies are small, and do not change the trends reported in this work. The QM/MM energies (*E*_QM/MM_) were calculated using Equation 1 as the separate energies of a QM calculation on the QM region (*E*_model, QM_) and an MM calculation on the complete system (*E*_real, MM_) minus an MM calculation on the QM region (*E*_model,MM_). QM/MM optimizations were initially done using DFT/AMBER with mechanical embedding included and were followed by DFT/AMBER single point calculations with electronic embedding included. We later also did full geometry optimizations with electronic embedding included at the DFT/AMBER level of theory.

(1) $$E_{\text{QM}/\text{MM}}=E_{\text{model},\text{ QM}}+E_{\text{real},\text{ MM}}-E_{\text{model},\text{ MM}}$$

### Mechanistic studies

**SCHEME 3:**
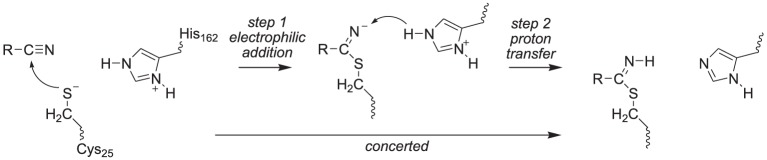
**Proposed reaction mechanisms of cysteine protease with nitrile-based inhibitors**.

The covalent inhibition of the inhibitor molecules was investigated via two possible scenarios, namely a concerted or a stepwise mechanism, Scheme 3. The first possibility is sequential nucleophilic addition and proton transfer in a stepwise mechanism, where the thiolate group of Cys_25_ attacks the carbon atom of the nitrile group first to form a covalent bond. Subsequently, the negatively charged nitrogen atom of this bond abstracts a proton from His_162_. Alternatively the nucleophilic addition and proton transfer happen in a single concerted reaction step. To ascertain the reaction mechanism of these inhibitors against cysteine protease enzymes we investigated both possible reaction mechanisms using two computational methods: Firstly, we did extensive density functional theory (DFT) studies on an active site model complex. Secondly, full quantum mechanics/molecular mechanics (QM/MM) studies on a substrate bound crystal structure were performed.

## Results and discussion

Thus, in the proposed reaction mechanism, the thiolate group of Cys_25_ reacts via a nucleophilic addition reaction with substrate to form a covalent linkage. The, thereby formed intermediate abstracts a proton from a nearby histidine residue (His_162_) to give the product complex, Scheme 2. Currently, it is unclear whether this mechanism is concerted or stepwise and what the effect of the protein is on the reaction mechanism.

**Figure 2:**
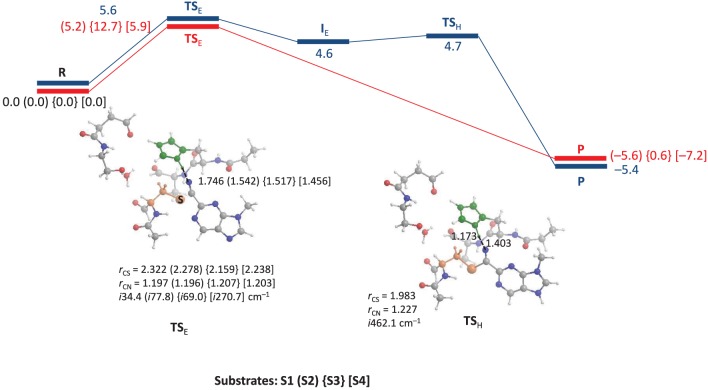
**QM-cluster calculations on the reaction mechanism of inhibition of substrate by cysteine protease**. In blue we give the stepwise mechanism and in red the concerted mechanism. Bond lengths are given in angstroms, the imaginary frequency in the transition states in cm^−1^ and relative energies (Δ*E*+*ZPE* with energies calculated with basis set B2) in kcal mol^−1^.

We started the work with a DFT investigation into the catalytic reaction mechanism of cysteine proteases, using QM-cluster model as described in Scheme 2 above and the results obtained with templates S1, S2, S3 and S4 are given in Figure 2. We define the reaction as proceeding from a reactant complex (R) that is initiated by an electrophilic attack of the thiolate group on the nitrile via a transition state TS_E_ leading to an intermediate state I_*E*_. In a subsequent proton transfer reaction via transition state TS_H_ we form the product complex P. Thus, using a substrate with the imidazolium group doubly protonated we find a stepwise mechanism, but if it is deprotonated on the N_*d*_ position a concerted pathway is found via a single transition state (TS_E_), where the imaginary mode gives simultaneous S–C bond formation and proton transfer from His_162_ to the nitrile nitrogen atom to form the product complex P. By contrast, for substrate S1 the reaction is stepwise via an intermediate complex and two barriers TS_E_ and TS_H_ separating the intermediate were characterized. As a consequence the processes for S2, S3 and S4 are concerted via a single transition state with an imaginary frequency that shows a mixture of both electrophilic attack and proton transfer. For inhibitor scaffolds S1, S2, and S3 this barrier is characterized by a small imaginary frequency, which is typical for sulfur-involved transition states (Kumar et al., 2011a). Using S4, the proton transfer is more dominating in the transition state, likely due to the later transition state and hence the imaginary frequency is larger (*i*270.7 cm^−1^).

Optimized geometries of the transition states (TS_E_ and TS_H_) are displayed alongside the mechanism of the reaction in Figure 2. The concerted TS_E_ transition states have the proton originating from His_162_ at a relatively short distance of 1.456–1.542 Å from the nitrile nitrogen atom, where it is 1.746 Å in the stepwise TS for substrate S1. Interestingly, the nitrile C–N distance shows little variation between the four different transition states and ranges from 1.196 to 1.207 Å. The C–S distance is slightly longer in the stepwise transition state as compared to the concerted transition states.

Energetically, the nucleophilic mechanisms are characterized by low energy barriers of between 5 and 6 kcal mol^−1^ for templates S1, S2, and S4, whereas S3 gives a somewhat enhanced barrier of 12.7 kcal mol^−1^ in the gas-phase. Therefore, the inhibition of cathepsin K by these nitrile-based inhibitors would be expected to be a fast and efficient process. Although technically the mechanism using substrate S1 is stepwise with well-characterized transition states for nucleophilic addition and proton transfer, the proton transfer barrier is very small (of the order of 0.1 kcal mol^−1^). This implies that the intermediate complex will have an ultrashort lifetime and proton transfer will happen instantaneously prior after the nucleophilic addition. Consequently, the mechanism perceived will be a concerted nucleophilic addition and proton transfer. Calculations with a dielectric continuum added that mimics a solvent gave little changes in the relative energies.

Replacement of one of the nitrogen atoms of the pyridine ring in S2 by a C–H group as in S3 leads to a raise in barriers for the nucleophilic reaction mechanism. Thus, using substrates S1, S2, and S4 the peptide chain N–H group of Cys_25_ interacts with the pyridine nitrogen atom in a weak hydrogen bonding interaction. However, replacement of this nitrogen atom by a C–H group leads to a repulsive interaction between the N–H and C–H groups, which leads to some tilting of the substrate group and weakening of the hydrogen bonding interactions between substrate and protein. Because of this the barriers are raised and unfavorable substrate inhibition is observed. This result is in good agreement with experimental inhibition studies that found higher activity for S2 type inhibitors than for pyridine containing inhibitors (Ehmke et al., 2012). The effect of substitution of the other pyridine nitrogen atom by C–H has little influence on the reaction mechanism and thermodynamics since it is not involved in direct hydrogen bonding interactions. Moreover, this group is located on the edge of the protein and points toward the solvent, where its thermodynamic perturbation will be limited. As such it may be difficult to establish the intricate details of the reactivity differences between S3 and S4 experimentally.

**Figure 3:**
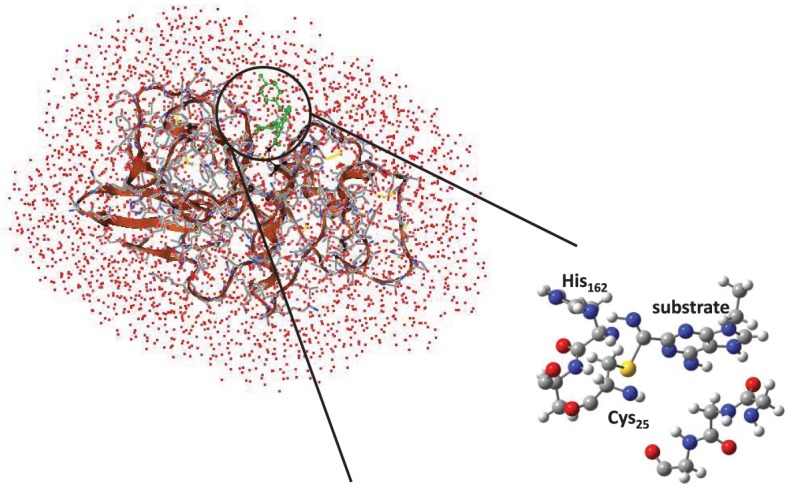
**QM/MM model and QM/MM active site as based on the 1U9V cathepsin K structure**.

To confirm the suggested mechanism and ascertain the effect of the protein, we decided to do a full QM/MM calculation on the mechanism using substrate S2. The cysteine protease active site is located on the surface of the protein, Figure 3, and the inhibitor forms a number of hydrogen bonds with the protein to maintain its binding mode. Inside the active site of the protein, the nitrile functionality reacts with Cys_25_, assisted by His_162_. Visualization of the protein structure identifies a covalent linkage between Cys_25_ and the substrate, hence is in a product-like P conformation.

**Figure 4:**
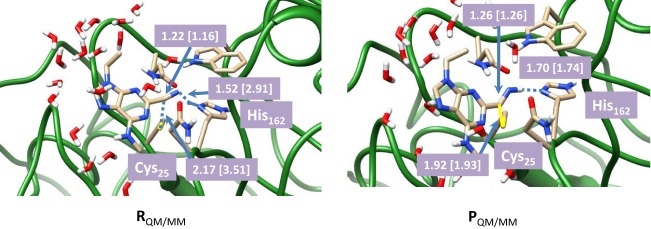
**Optimized geometries with bond lengths in angstroms of reactant and product geometries for nitrile-based inhibitors of cathepsin K as calculated with QM(B3LYP/6-311G)/MM**. Data in square brackets were obtained with electronic embedding included.

To understand the effect of protein and solvent on the mechanism of these inhibitors we decided to do a full QM/MM study starting from the described crystal structure. We initially optimized the reactant (R) and product (P) geometries using our QM/MM model and extracts of these structures are given in Figure 4. We used two procedures for the geometry optimizations: (*i*) with mechanical embedding included and (*ii*) with electronic embedding included. Thus, it occasionally happens that structures and consequently relative energies are dependent on the full environmental perturbations of the whole protein on the active site and with electronic embedding these interactions are included in full. The reactant complex has the inhibitor in a conformation where the nitrogen atom is in hydrogen bonding distance to the proton of the imidazole group of His_162_ at a relatively short distance of 1.519 Å. Hence the inhibitor molecule will be strongly bound in the substrate binding pocket in a specific orientation. The sulfur atom of the cysteine residue is located at the relatively large distance of 2.166 Å to the carbon atom of the nitrile. This geometry changes somewhat when electronic embedding is included and the histidine moves to a position in hydrogen bonding distance to the thiolate of Cys_25_, which results in somewhat larger distance away from the substrate. In the mechanically embedded structure the thiolate also forms a hydrogen bonding interaction with the peptide backbone N–H of Ala_161_ (2.488 Å) as well as a weak hydrogen bond with the hydrogen atom of the H–C^α^ atoms of His_162_ at a distance of 2.636 Å. In the product complexes these hydrogen bonding interactions are lost and the peptide chain containing the Ala_161_–His_162_ residues has moved away from the Cys-inhibitor linkage. Thus the nitrogen atom of the imidazole group of His_162_ has formed a weak hydrogen bonding interaction with one of the hydrogen atoms of the CH_2_ group of Cys_25_ instead (distance of 2.227 Å). By contrast, the thioester group of Cys_25_ interacts with the H–C^α^ group of His_162_ (distance of 2.830 Å) in the product complexes, and consequently considerable changes in the protein have occurred during the reaction process. As a result of this the relative energies for the potential energy profile has undergone some drastic changes.

The calculated reaction energy for the process from R_QM/MM_ to P_QM/MM_ using mechanical embedding only is −20.0 kcal mol^−1^, which in comparison to the QM-cluster calculations described above, is indicative of an irreversible process. However, it is known from experiment that the reaction is reversible, which means that the energeties calculated with mechanical embedding are not realistic. In particular, enzymatic studies on this system have shown that covalent binding of the inhibitor is a reversible process. The calculations with mechanical embedding included, therefore, are a poor representation of the actual inhibition process. To find a more realistic pathway, we subsequently recalculated the QM/MM calculations with electronic embedding included. As will be shown in the next paragraphs these calculations predict an almost thermoneutral reaction mechanism and consequently, a reversible reaction mechanism in agreement with experiment. Most probably the polarity of the inhibitor binding site and the enzyme makes it essential to include full electronic embedding in the calculations.

**Figure 5:**
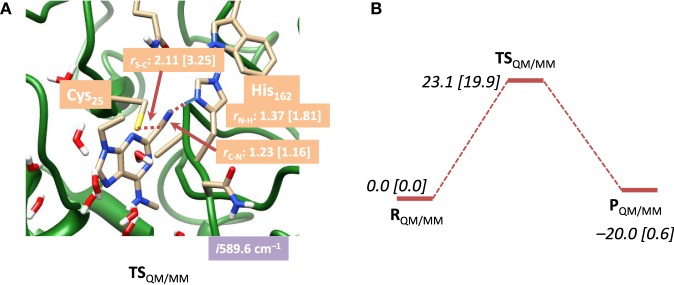
**(A)** Optimized geometry with bond lengths in angstroms of TS_QM/MM_ for nitrile-based inhibitors in cathepsin K as calculated with QM(B3LYP/6-311G)/MM. **(B)** Potential energy landscape from reactants to products as obtained with QM/MM. Energies are in kcal mol^−1^ and contain ZPE corrections. Values in square brackets were obtained with electronic embedding included.

In a subsequent set of calculations we explored the potential energy profile between reactants and products in detail using QM/MM with both mechanical and electronic embedding models. To this end we initially did a full geometry scan, whereby the C–S bond formation was kept fixed at specific distances while we optimized all other degrees of freedom starting from R_QM/MM_. In addition, we did geometry scans in the reverse direction starting from P_QM/MM_ for the proton transfer back from the CN–H group to His_162_. These geometry scans gave insight onto the potential energy profile of the reaction and the maxima from the scans were used as starting points for the transition state searches. Our optimized transition state geometries and potential energy landscapes are shown in Figure 5. The barrier is characterized by a large imaginary frequency of *i*589.6 cm^−1^ that implicates the proton transfer from His_162_ to the nitrile group. It is also substantially higher in energy than the one calculated for the DFT model above and we find it 23.1 kcal mol^−1^ in energy above the reactants using the mechanical embedding procedures. Geometrically, the C–S distance is in between that found for reactants and products. Moreover, an attempt to optimize a local minimum and a subsequent C–S bond formation transition state failed and led to reactant structures in all cases. Therefore, the reaction proceeds in a concerted mechanism via a transition state that is dominated by the proton transfer motion. Using electronic embedding corrections the barrier height drops slightly to 19.9 kcal mol^−1^. Therefore, electronic embedding has a small barrier-lowering effect on the calculations. The nucleophilic addition barrier calculated with QM/MM is in perfect agreement with that found for native cysteine proteases, for which a value of 19.8 kcal mol^−1^ was calculated (Ma et al., 2007). Therefore, the electronic embedding corrections appear to have a large effect on the overall thermodynamics of the reaction but only a minor effect on the kinetics. The electronically embedded reaction energy is close to thermoneutral and very similar to that found for the QM-cluster results described above. Experimentally, it has been shown that the reaction is reversible, which implicates an almost thermoneutral reaction mechanism, in agreement with our QM-cluster and QM/MM with electronic embedding results. Clearly, the mechanical embedding QM/MM calculations overestimate the overall thermodynamics of the reaction, although the kinetics is reasonable.

**Figure 6:**
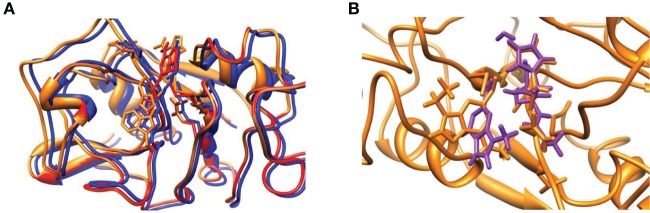
**(A)** Overlay of optimized geometries of R_QM/MM_ (red), TS_QM/MM_ (amber) and P_QM/MM_ (blue) structures. **(B)** Overlay of TS_QM/MM_ (amber) and TS_E,S1_ (violet). QM/MM structures obtained with mechanical embedding only.

In order to understand the large differences and reaction energetics between the DFT model study and the QM/MM results, we investigated structural differences between the optimized geometries first, see Figure 6. Panel Figure 6A displays an overlay of the three QM/MM optimized geometries. Despite the fact that these structures have resulted from a full geometry optimization without constraints, actually the peptide chain is virtually in the same position for all geometries. In the active site, there are some obvious structural differences due to formation of a linkage between Cys_25_ and the inhibitor, which is absent in the reactant. This bond formation changes the position of the histidine side-chain as well as other proton donors/hydrogen bonding groups to the active site, including the Trp_184_ and Gln_19_ residues.

The overlay of the two transition state structures for substrate S1 activation as obtained by DFT and QM/MM is given in Figure 6B. Even though the DFT model complex had four fixed carbon atoms in positions of the original protein structure, the full geometry optimization of the reaction mechanism led to a structure that significantly deviated from the actual enzyme conformation. Thus, the overlay of TS_QM/MM_ and TS_E, S1_ shows a twist of the substrate by almost 90° as well as reorientation of the protein backbone. In the model complex, there is sufficient geometric flexibility so that the system will relax to its most ideal configuration. In the enzyme, by contrast, steric interactions of the protein structure prevent reorientation of the geometry and the most ideal configurations as those found for the DFT model complexes are not stable. Because of that the energetics of the potential energy profile has shifted dramatically and the nucleophilic addition step is raised in energy. This is indeed what was found on native cysteine proteases, where it was shown that disruption of the hydrogen-bonding network led to proton affinity differences of the histidine and cysteine groups (Mladenovic et al., 2008b). Based on these studies Engels et al. predicted enhanced nucleophilic addition reactivity upon removal of the hydrogen-bonding network. Indeed that is what we find when we compare the studies of the DFT model complex (without the water layer) and the QM/MM result. Recent work of ours on the reactivity of iron(IV)-oxo and manganese(IV)-oxo intermediates in aliphatic reaction mechanisms also showed reduced reactivity for complexes whereby the active oxidant underwent hydrogen bonding (accepting) interactions so that this may be a general feature in enzymatic reaction mechanisms (Latifi et al., 2013).

## Conclusions

We report a combined DFT and QM/MM study into the mechanism of inhibition of nitrile-based inhibitors to cathepsin K. We show here that a DFT model complex gives dramatic differences in optimized geometries and relative energies as compared to QM/MM. We show that despite geometric constraints the DFT structure has undergone dramatic changes with respect to its original conformation and does not fit the enzymatic pocket anymore when an attempt is made to reinsert the structure into the protein. These changes affect the relative energies of the reaction. All calculations predict a concerted nucleophilic addition reaction with simultaneous C–S bond formation and proton transfer. We also did QM/MM calculations and show that a procedure with full electronic embedding included is necessary to describe an irreversible reaction process. This again highlights the importance of model and method choice in quantum chemical calculations.

### Conflict of interest statement

The authors declare that the research was conducted in the absence of any commercial or financial relationships that could be construed as a potential conflict of interest.
